# Dr. Forbes on the Climate of Penzance and District of the Land's End

**Published:** 1821-03-01

**Authors:** 


*Observations on the Climate of Penzance and the District of the Land's End in Cornwall; with an Appendix, containing Meteorological Tables, and a Catalogue of the rarer indigenous Plants. Read before the Penwith Agricultural Society, and published by Request of the Members.* By John Forbes, M. D. Secretary of the Royal Geological Society of Cornwall, and Physician to the Penzance Dispensary. Octavo, sewed, pp. 64. London, 1821.

It is highly desirable that, before we order our invalids to a foreign climate, we should be acquainted with our own. Dr. Forbes has set a very good example in exploring the medical topography of a favoured spot in this island, which, all things considered, may probably vie, in no small degree, with some of those boasted cisalpine, or even transalpine retreats where Hygeia is supposed to hold her court, beneath a cloudless sky, and breathe recruiting health on every zephir. The more intimately we become acquainted with the face of Nature around us, the more we will be convinced of the general impartiality with which our Creator has scattered his gifts for the use of mankind. To the Laplander and the Æthiopian, the Hindoo and the Peruvian, the sun appears, for precisely the same number of hours, above the horizon, in the course of the year, however various may be the length of their days and nights. And if this great enjoyment is so equally distributed in reality, though so partially in appearance, we may fairly conclude that, in other things also, the equilibrium is far greater than superficial observation is disposed to allow.

Cornwall in general, but the district of the Land's End in particular, possesses all the advantages and disadvantages of a small island moderately elevated above the level of the sea, and placed thirty or forty miles to the westward of the most southerly point of the main land.

I. *Temperature.* From a great number of authentic documents, and accurate thermometrical observations, Dr. Forbes is disposed to place the mean temperature of this place at 52°. But, like all small islands, or in other words, like the ocean itself, Penzance has a mean summer temperature considerably *under*, and a mean winter temperature considerably *above*, the mean of places similarly situated as to latitude, but placed at a distance from the sea. This appears evident from the following table of mean annual temperature, given in round numbers.

| Jan. | Feb. | Mar. | Apr. | May. | June. | July. | Aug. | Sep. | Oct. | Nov. | Dec. |
|---|---|---|---|---|---|---|---|---|---|---|---|
| 41° | 44° | 44° | 49° | 56° | 60° | 62° | 61° | 58° | 53° | 46° | 43° |

| Spring. | Summer. | Autumn. | Winter. |
|---|---|---|---|
| 45° | 59° | 57° | 43° |

The *equability* of temperature, in Penzance, from its peninsular situation, exceeds that of any other place in this island—an equability no less conspicuous in the variations of temperature of successive days, or hours, than in the extreme range of the day, month, or year, as may be seen in table No. 1. The mean variation of the thermometer in successive days, has been as follows:—

| Winter. | Spring. | Summer. | Winter. |
|---|---|---|---|
| 3°.77. | 2°.97. | 1°.72. | 2°.28. |

"In regard to the comparative temperature of the different seasons and months, it appears that this is equally indicative of the uncommon equability of the climate of this place.

"The greatest variation between the mean temperature of any two successive months is 7°; while the mean variation of successive months is only 3.5°, or, calculating from the morning observation, 3.33°. The following is the statement of this mean monthly variation for the different seasons :—

| | Winter. | Spring. | Summer. | Autumn. |
|---|---|---|---|---|
| By the morning observation - - | 3.66° | 2° | 4.66° | 3° |
| —— mean of morn. and aftern. | 4° | 2.66° | 4.66° | 3.33° |
| —— — morn. aftern. and even. | 4° | 2.66° | 4.33° | 3° |

"The variation of temperature between the different *seasons* is also very small; in other words, their mean temperature is remarkably equal. The following is the difference:—

| | | |
|---|---|---|
| Between Winter and Spring | - - - - | 2° |
| ——— Spring and Summer | - - - - | 14° |
| ——— Summer and Autumn | - - - | 2° |
| ——— Autumn and Winter | - - - | 14° |

"The coldest month is January;—the warmest month is July:—April and October approach nearest to the mean temperature of the year." P. 10.

From the foregoing statements, as our author observes, it is evident that the temperature of Penzance is remarkable for its small annual, monthly, and daily range, and for its equability from day to day. The mean temperature of its winters is uncommonly high, for a British locality, and its summer heat uncommonly low for its southerly latitude. The difference of temperature between the different seasons is remarkably small—especially between Autumn and Winter, and between winter and spring.

Thus at Sidmouth the mean temperature for two years of November was 44°. December 41°. January 34°. While that of Penzance, in the same months was as follows: Nov. 45°.—Dec. 49°—Jan. 37°; making a general superiority in Penzance of 4°; and in the month of Dec. of 8°. The difference between Penzance and Exeter, Plymouth, Clifton, Isle of Wight, Gosport, London, &c. is also remarkable, as deduced from authentic tables. During the severe weather of January 1820, when the thermometer, at 8 o'clock in the morning, stood at 11°. in London, it stood at 22° in Penzance, at the same hour. "The lowest point to which it has fallen, at Penzance, at 8 o'clock in the morning, during the last fourteen years, has been 19 , and that only once." The greatest height which the thermometer reached at Penzance during fourteen years was 78°, whereas it is not uncommon, in other parts of England, to reach from 80 to 90 in some hot days. In illustration of the *equability* of the Penzance climate, Dr. Forbes gives a parallel between it and Edmonton, near London, for the month of August, 1820.

| | | London. | | Penzance. |
|---|---|---|---|---|
| "Absolute maximum | - - - | 81° | — | 67° |
| ———minimum | - - - - | 38 | — | 46 |
| Mean of the maxima | - - - - | 72 | — | 46 |
| ——— minima | - - - - | 50 | — | 54 |

| | | London. | | Penzance. |
|---|---|---|---|---|
| Mean of both observations | - - | 61° | — | 59° |
| Extreme monthly range | - - - - | 43 | — | 21 |
| Diurnal range | Max. - - - - | 32 | — | 14 |
| | Min. - - - - | 11 | — | 5 |
| | Mean - - - - | 21 | — | 9" P. 17. |

After introducing various tables in elucidation of the meteorology of Penzance, as far as regards temperature, Dr. Forbes concludes in the antiquated but racine terms of old Carew.

"The *spring* visiteth not these quarters so timely as the eastern parts. *Summer* imparteth a very temperate heat, recompensing his slow fostering of the fruits with their kindly ripening. *Autumn* bringeth a somewhat late harvest, especially to the middle of the shire, where they seldom inn their corn before Michaelmas. *Winter*, by reason of the South's near neighbourhood, and sea's warm breath, favoureth it with a milder cold than elsewhere, so, as upon both coasts, the frost and snow come very seldom, and make a speedy departure." 22.

II. *Atmospheric Pressure.* The locality of Penzance, the humidity of its climate, and the variability of its winds, would lead to the expectation of peculiarity in the barometrical phenomena. Yet the only peculiarities observable are, the inferior altitude and smaller range of the mercurial column. The mean height of the barometer is 29.64, and the mean annual range 1.96. The mean monthly range is 1.07.

III. *Humidity.* Cornwall has always been considered a wet climate. This is true as regards hygrometrical humidity and the number of days on which rain falls; but as regards the actual *quantum* of rain, the imputation is exaggerated. Fogs are by no means common, yet the warm westerly winds bring with them a sort of drizzly rain, sufficient to wet thoroughly grass and other vegetables, or the clothes of a person exposed; while neither the rain-guage, nor the roads or streets shew any indication of its presence. The number of days on which rain falls is computed at 157; yet the following remark of Dr. Borlase is peculiarly applicable.

"Our rains in Cornwall are rather frequent than heavy or excessive; and we have seldom a day so thoroughly wet but that there is some intermission, nor so cloudy but the sun will find a time to shine." 26.

The average number of days on which snow falls, in any one year, is little more than *two and a half;* and in the last fourteen years, there have been four in which snow did not fall at all. It seldom remains on the ground more than a few hours.

IV. *Winds.*—Cornwall is as remarkable for the variability of its winds, as for the steadiness of its temperature. Old Carew draws a quaint but faithful picture of this feature in the climate.

"Touching the temperature of Cornwall," says Carew, "the air thereof is cleansed as with bellows, by the billows, and flowing and ebbing of the sea, and therethrough becometh pure and subtle, and by consequence healthful." "This notwithstanding" (he says in another place) "the country is much subject to storms, which fetching a large course in the open sea, do from thence violently assault the dwellers at land, and leave them uncovered houses, pared hedges, and dwarf trees as witnesses of their force and fury." 34.

Our limits prevent our noticing numerous other curious and interesting subjects in this pamphlet; yet we hope we have exhibited enough to convince our brethren, that in Cornwall they have no mean substitute for the climate of Italy and the South of France, with JOHN BULL'S FIRE-SIDE into the bargain—a fire-side around which more real comforts are concentrated than in any other country in the world.

"I shall subjoin a table of the mean morning temperature of the three coldest months at Nice, Pisa, and Rome, (extracted from Dr. Clark's late excellent work on the climate and diseases of France and Italy,) compared with that of Penzance for the same years. This statement will show the difference of temperature between these places and Penzance to be much less, I think, than might have been anticipated from considering the difference of geographical position; certainly much less than had been conceived by myself before examination.

| Farenheit's Thermometer | Time of observation | Dec | Jan | Feb | Mar |
|---|---|---|---|---|---|
| Nice, 3 years, (1815-1817) | sun-rise | 44 | 44 | 47 | 45 |
| Pisa, 3 years, (1814-1816) | sun-rise | 42 | 40 | 43 | 41 |
| Rome, 3 years (1815-1817) | 7 a. m. | 42 | 41 | 43 | 42 |
| Penz. 3 years, (1815-1817) | 7 & 8 a. m. | 42 | 41 | 44 | 42 |

64.

The foregoing paper sufficiently evinces the accurate observations and legitimate deductions of its very able author; and we hope that Dr. Forbes will follow up his investigation of the medical topography of Cornwall, by observations on the diseases to which the climate is favourable—and especially on that great enemy of England, PULMONARY CONSUMPTION.

